# Reversible electroadhesion of hydrogels to animal tissues for suture-less repair of cuts or tears

**DOI:** 10.1038/s41467-021-24022-x

**Published:** 2021-07-20

**Authors:** Leah K. Borden, Ankit Gargava, Srinivasa R. Raghavan

**Affiliations:** Department of Chemical & Biomolecular Engineering, University of Maryland, College Park, MD USA

**Keywords:** Biomaterials, Biomedical materials, Soft materials

## Abstract

Electroadhesion, i.e., adhesion induced by an electric field, occurs between non-sticky cationic and anionic hydrogels. Here, we demonstrate electroadhesion between cationic gels and animal (bovine) tissues. When gel and tissue are placed under an electric field (DC, 10 V) for 20 s, the pair strongly adhere, and the adhesion persists indefinitely thereafter. Applying the DC field with reversed polarity eliminates the adhesion. Electroadhesion works with the aorta, cornea, lung, and cartilage. We demonstrate the use of electroadhesion to seal cuts or tears in tissues or model anionic gels. Electroadhered gel-patches provide a robust seal over openings in bovine aorta, and a gel sleeve is able to rejoin pieces of a severed gel tube. These studies raise the possibility of using electroadhesion in surgery while obviating the need for sutures. Advantages include the ability to achieve adhesion on-command, and moreover the ability to reverse this adhesion in case of error.

## Abstract (web summary)

The authors demonstrate strong adhesion of cationic hydrogels to bovine tissues under a DC electric field. Such electroadhesion can be reversed by switching the polarity of the field. This approach could enable simpler surgeries, where sutures are not needed.

## Introduction

The phenomenon of “electroadhesion” involving two oppositely charged polyelectrolyte hydrogels was first reported about 10 years ago^1^. The starting point is to take two solid gels (slabs or strips), each formed by chemical crosslinking of monomers, with one gel having a cationic backbone and the other an anionic backbone. The two gels are contacted with each other along one face and electrodes are placed along either side, as shown by Supplementary Movie 1. Thereafter a DC voltage is applied in a specific orientation. As shown in the movie, within seconds, the two gels become strongly adhered. The movie also shows that the same gels will not adhere if contacted in the absence of the field. Thus, the adhesion is induced by the electric field, and hence the term ‘electroadhesion’ for this phenomenon^1–8^. If the polarity of the field is reversed, the gels lose their adhesion and can be detached (this too is shown by the movie). The mechanism for electroadhesion is still not completely understood, but it is believed to involve molecular rearrangement of both polymer chains and counterions at the gel-gel interface^1–8^. Thus far, there have been only a few applications of electroadhesion, such as to assemble gels into 3-D structures^6–8^. On the whole, however, electroadhesion has remained an oddity that has attracted only moderate interest in the scientific community.

In this study, we hypothesized that electroadhesion could be induced between hydrogels and other kinds of soft matter. In testing this hypothesis, we have discovered that gels could indeed be electroadhered to animal (bovine) tissues. This result is surprising because, while some tissues can be soft and gel-like, they are structurally very different from conventional polymer gels^9,10^. We show that gel-tissue electroadhesion only works between certain types of gels and tissues, and the reasons for the same are discussed. Our work significantly enlarges the landscape of materials that can be electroadhered, and thereby the utility of this phenomenon. One obvious application is as an adhesive to reseal damaged tissues. Currently, if a tissue is torn, sutures or staples are needed to rejoin the torn pieces and thereby allow the tear to repair naturally over time. This suturing is a surgical operation that requires considerable skill on the part of a surgeon^11^, and this often implies a difficult and expensive procedure. Adhesives have been explored as alternatives to sutures during surgery^11–18^. Several polymeric adhesives are available for surgical use, including those based on cyanoacrylates, fibrin, and polyethylene glycol (PEG) derivatives^12^. Most of these materials are intrinsically sticky and cling upon contact with tissue. Such adhesives have many limitations: in particular, they are usually not strong enough to hold two cut pieces of tissue together. As a result, adhesives usually cannot replace sutures, but are sometimes used along with sutures (e.g., instead of ten sutures, a combination of two sutures and an adhesive may be used)^16,17^. Also, if the adhesive forms a solid film (immediately after application, or after a period of drying), this could result in a physical barrier that hinders the supply of nutrients to the underlying tissue^19^. In comparison, adhesives in hydrogel form are preferable due to their soft nature and their permeability to water and nutrients^11–15^. For a gel-adhesive to provide a viable alternative to sutures, it should stick strongly to tissues. Even better would be to have a non-adhesive gel that can develop adhesion on command, and moreover, for this adhesive to be reversible (removable) in case of error.

To test the hypothesis that electroadhered gels could potentially enable an alternative mode of surgery, we describe a series of in vitro studies in this paper. First, we describe model systems of oppositely charged polymer gels, one in the form of rectangular strips and the other as hollow tubes. In an initial case, a hole is made in the tube wall and a small gel strip is electroadhered over the hole. We show that water can be flowed through the patched tube (with no leaks through the sealed hole) at pressures that exceed normal blood-pressure. Next, in a more extreme case, the tube is cut into two and we attempt to join the segments by adhering a sleeve of gel around the cut segments. For this, a long gel strip that is robust and flexible is fabricated, and by electroadhesion, we are able to effectively “suture” the cut segments of the tube. We then describe similar experiments on the use of electroadhered gels to seal holes in a tubular animal tissue, i.e., a section of bovine aorta. By applying a DC field of 10 V for a short time (10–20 s), strong adhesion between gel and tissue is achieved. This adhesion can be reversed at a later time by reversing the polarity of the field. Our studies collectively demonstrate the potential utility of electroadhesion in biomedical applications.

## Results and discussion

### Gel-Gel electroadhesion

**Fig. 1:**
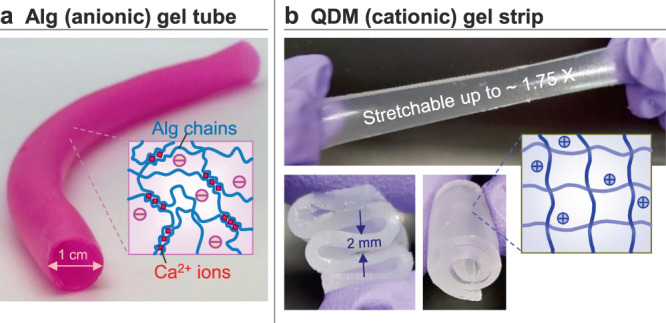
Gels used in our electroadhesion studies. **a** Anionic gel of alginate (Alg), crosslinked by divalent Ca^2+^ cations. The gel is made in the form of a hollow tube. **b** Cationic QDM gel strip, made by polymerization of acrylamide derivatives. The photos show that the gel is elastic, stretchable and flexible. Schematics of the gel structure are shown as insets in each case.

Our initial studies were conducted with a combination of gels, one cationic and the other anionic. To mimic tubular tissues, we fabricated the anionic gel in the form of a tube. The gel in this case is composed of the anionic polysaccharide sodium alginate crosslinked into a network by divalent Ca^2+^ cations. The procedure for creating tubes with a wall of alginate (Alg) gel was described in an earlier study from our lab^20^ and is adapted here (see “Methods” section for details). Through this procedure, we can control all the dimensions of the tube, including the length, inner diameter, and wall thickness. Figure 1a shows a 10-cm long tube with an inner diameter of 1 cm and a wall thickness ~1 mm. The inset illustrates the structure of the gel wall, which consists of Alg chains connected at zones by Ca^2+^ ions. The tube has a pink color due to a trace amount of rhodamine B (RB) dye added during the synthesis.

The counterpart to this tube is a cationic gel, made in the form of a rectangular strip. This gel is synthesized by polymerizing a mixture containing acrylamide (AAm, a nonionic monomer), quaternized dimethyl aminoethyl methacrylate (QDM, a cationic monomer), bis(acrylamide) (BIS, a nonionic crosslinker) and laponite (LAP) nanoparticles. The molar ratio of QDM relative to all the monomers dictates the level of charge on the gel strands, and this is kept at 16 mol%. The ratio of BIS to all the monomers dictates the stiffness of the gel, and this is maintained at 1.6 mol%. If the BIS content is too high, the gel becomes brittle. We found that adding 0.1 wt% of LAP to the gelling mixture significantly improves the flexibility and stretchability of the final gel, consistent with previous studies from our lab^21^. The overall gel is denoted as QDM to signify its cationic nature. Figure 1b shows that the QDM gel strip is flexible enough to be twisted or rolled up. The strip can also be stretched up to ~1.75 times its original length without rupture.

**Fig. 2:**
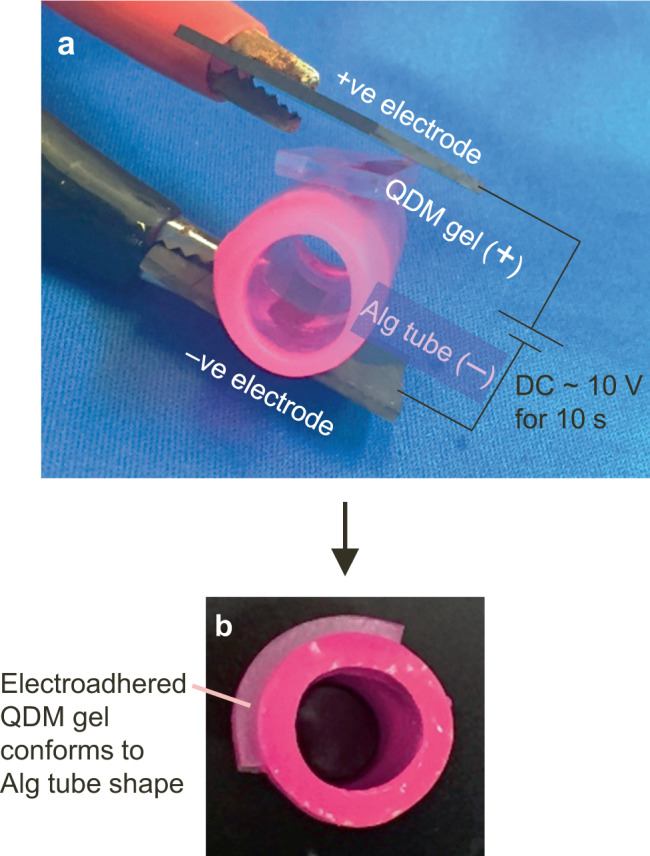
Electroadhesion of QDM gel-strip to Alg tube. **a** The gel and tube are contacted with graphite electrodes, with the positive electrode touching the cationic QDM gel and the negative electrode touching the anionic Alg tube. **b** Upon applying 10 V of DC for 10 s, the gel gets tightly adhered to the tube and conforms to the tube shape. In cases where a puncture is made in the tube wall, adhesion of the gel over the puncture location serves to patch up the puncture (see next figure).

We first confirmed that the cationic QDM gel-strips could be electroadhered to the anionic Alg gel-tubes. Our setup for electroadhesion involves two graphite electrodes and a DC power supply. The electrodes have to be placed in contact with the above gels in a particular orientation, as shown by Fig. 2a. That is, the strip and tube are brought into contact, and then the positive electrode (E^+^) is made to contact the cationic gel strip (G^+^), while the negative electrode (E^−^) is contacted with the anionic gel tube (G^−^). With this orientation (denoted from now on by E^+^G^+^G^−^E^−^), a DC voltage of 10 V is applied for ~10 s. When the voltage is switched off, the QDM strip is found to be strongly adhered to the alginate tube (Fig. 2b), and this adhesion persists thereafter. If the reverse electrode orientation (E^+^G^−^G^+^E^−^) is used at the start, the two gels will not stick. Conversely, if electroadhered gels are reconnected to the field in the above reverse orientation, and a 10 V field is applied for ~10 s, the gels lose their adhesion and can be easily detached. These features are similar to those illustrated in Supplementary Movie 1. To our knowledge, this is the first demonstration of electroadhesion between a covalently crosslinked gel and a physically crosslinked one. In previous reports of electroadhesion^1–8^, the gels were all covalently crosslinked.

### Electroadhesion for repairing cut or broken gel-tubes

In our first set of experiments, we introduced a cut in the wall of an alginate tube and stuck a QDM gel over the cut. The cut was made with a needle or razor blade and its size could be varied. We then made rectangular patches of the QDM gel (15 mm long, 8 mm wide and 2 mm in thickness). Using the electroadhesion procedure described above, we affixed the QDM gel patch so as to cover the cut in the tube wall. Note that the patch adheres tightly to the tube and it conforms to the tube’s curvature (Fig. 2b).

**Fig. 3:**
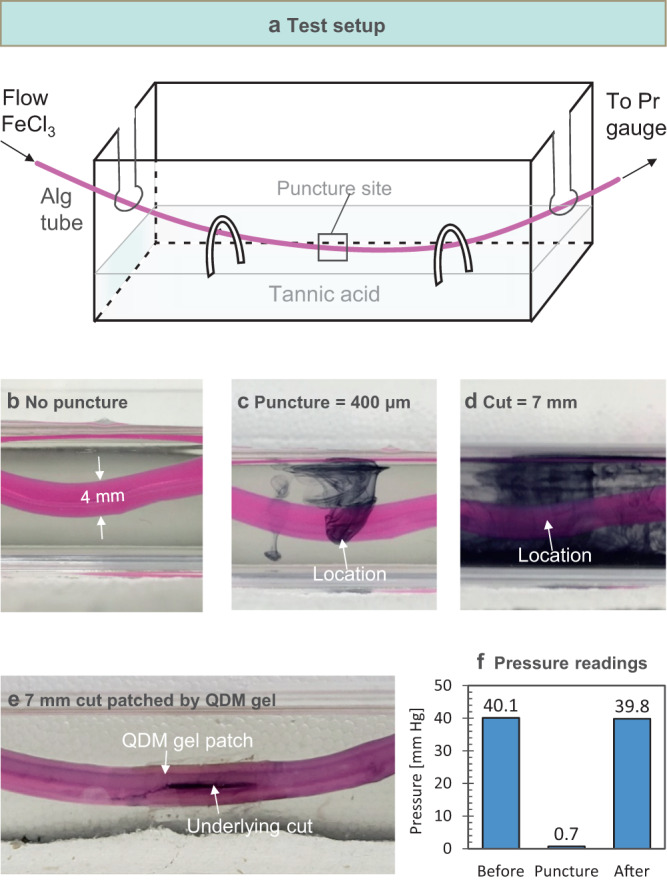
Electroadhesion of QDM gel to patch a cut in the Alg tube wall. **a** Schematic of test setup. An aqueous solution of 0.1 wt% FeCl_3_ is pumped through the lumen of the Alg tube, which is submerged in an aqueous bath of 0.1 wt% tannic acid. If the FeCl_3_ leaks out of the tube, it reacts with the tannic acid and a black precipitate is formed immediately in the bath. **b** When the tube is intact, there is no leakage and the bath is clear. **c** Tube is punctured with a needle to create a hole of 400 µm diameter. **d** Tube is cut to a length of 7 mm with a blade. In (C) and (D), as the fluid in the tube leaks out, the black precipitate can be seen in the bath. **e** The tube from (**d**) is patched by a QDM gel, and when flow is resumed through the tube, no leakage can be seen. **f** A pressure gauge placed upstream of the tube records the pressure in the tube. The pressure drops to near-zero in the case of a cut in the tube (similar to (**d**)) as the fluid leaks out. When the tube is patched up (similar to (**e**)), the pressure is restored to its original value.

To test the strength of the patch-tube adhesion, we introduced a flow of fluid through the patched tube. If the patch was not affixed, fluid would leak out of the cut in the tube. The question then is whether the patch could completely seal the leak and moreover if it could withstand the pressure exerted by the fluid. We developed a protocol for leakage tests that involves submerging the Alg tube in a water bath containing 0.1% of tannic acid (Fig. 3a, b). A 0.1 wt% solution of iron chloride (FeCl_3_) in water is then flowed through the lumen of the tube using a peristaltic pump. When FeCl_3_ contacts tannic acid, a black precipitate of ferric tannate is instantly formed^22^. Even if a small puncture (400 µm) is made in the tube wall using a needle, the leakage of fluid from the puncture can be readily detected by the eye due to the formation of the black precipitate (Fig. 3c). This leakage is much greater when a large cut (7 mm in length) is made in the tube wall with a blade (Fig. 3d). Figure 3e shows the Alg tube with the above large cut patched by a QDM gel using electroadhesion. In this case, there is stable flow of fluid through the tube with no leak whatsoever.

To quantify the above experiment, we installed a pressure gauge to the tube upstream of the puncture site. This measures the pressure *P* exerted by the fluid flow on the tube wall. When some of the fluid leaks out through the cut, *P* drops relative to its initial value (which corresponds to stable flow with no leak). If the leak is considerable, then *P* drops to nearly zero. For example, the bar graph in Fig. 3f shows that the initial *P* is 40.1 mm-Hg as fluid is flowed through the tube at a flow rate of 5 mL/min. When a cut of 7 mm length is made in the tube wall and flow is resumed at the above flow rate, *P* drops to 0.7 mm-Hg. Next, the cut is patched with a QDM gel using electroadhesion and the experiment is repeated—*P* then increases to 39.8 mm-Hg, which is nearly the same as the initial pressure. These data show that the electroadhered patch holds up well to the above flow conditions.

The pressure readings above depend on both the flow conditions and the size of the cut in the tube^23^. If the flow rate is increased above a critical value, the pressure exerted by the fluid is able to dislodge the electroadhered patch and the fluid then leaks into the surrounding bath. We define the pressure at this point of failure as the burst pressure *P*_burst_, and it represents a limit for the conditions studied. Supplementary Fig. 1 shows pressure readings for various puncture/cut sizes. *P*_burst_ is 252 mm-Hg for a small (0.4 mm) puncture, 216 mm-Hg for a medium (1.4 mm) puncture, and 82 mm-Hg for a large (7 mm) cut. These *P*_burst_ values indicate robust sealing capability under typical blood-flow conditions (normal systolic blood pressure being 120 mm-Hg in healthy humans)^24^. Note that *P*_burst_ can be easily increased by either using a larger gel-patch around the cut or by introducing a second gel-patch over the first in a cross-geometry.

**Fig. 4:**
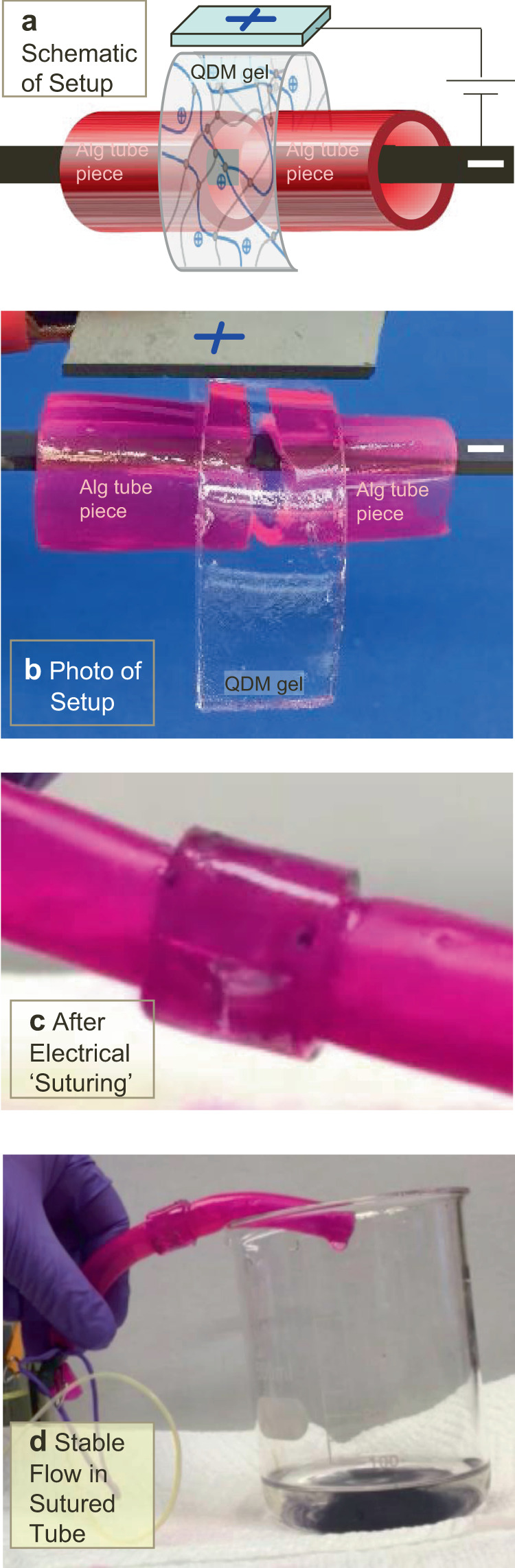
Electrical “suturing” of two severed segments of a tube. A long QDM gel-strip is used as a sleeve around the two pieces of the Alg tube. The electrode orientation is as indicated. Schematic of the process is shown in (**a**) and a photo in (**b**). **c** Following this process, the Alg tube segments are found to be ‘sutured’ (joined) by the gel sleeve. **d** Stable flow occurs through the repaired tube to the waste beaker. The entire sequence of events is demonstrated by Supplementary Movie 2.

Next, we examined if electroadhesion could repair a much more extreme “injury” compared to a cut in the tube wall. In this case, we severed the alginate tube in half and attempted to join the two pieces using a QDM gel-strip. First, a long and flexible gel-strip (15 mm long, 8 mm wide and 2.5 mm in thickness) was made. The two pieces of the tube were contacted laterally and the QDM gel-strip was wrapped around the tube segments (Fig. 4a, 4b). During electroadhesion, the negative electrode was kept in contact with the tube at all times while the positive electrode was rotated along the exterior of the gel-strip (Fig. 4b). This process is also shown by Supplementary Movie 2. The net result is that the QDM gel functions as a sleeve that wraps around the cut pieces (Fig. 4c). Note that the length of the gel strip was chosen to match the perimeter of the sleeve so that there is no gap between the ends of the strip. Thus, the patched tube behaves like a single entity. We can flow fluid through the patched tube without leaks (Fig. 4d), and this is also shown by Supplementary Movie 2.

### Gel-tissue electroadhesion

**Fig. 5:**
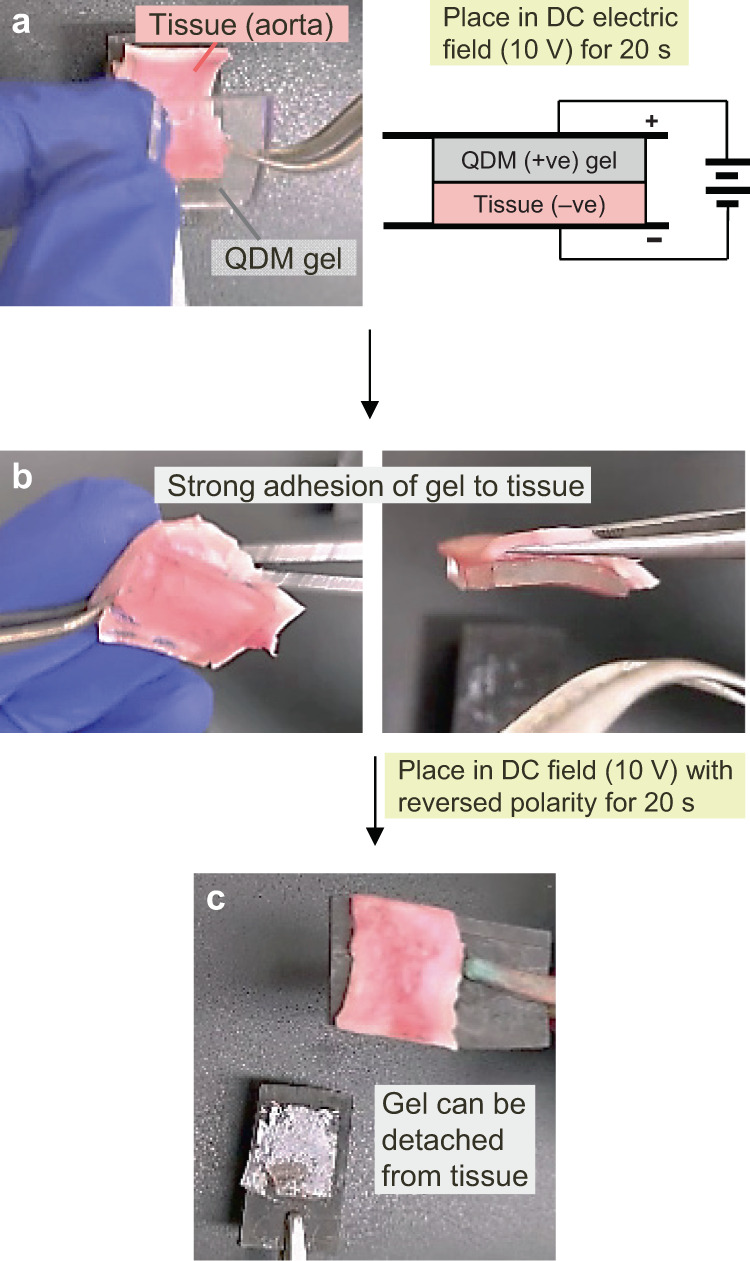
Electroadhesion of QDM gel to bovine tissue. **a** A strip of tissue (T^−^), specifically bovine aorta, and a strip of cationic QDM gel (G^+^) are contacted in a E^+^G^+^T^−^E^−^ configuration, with the gel touching the positive and the tissue the negative electrode. 10 V of DC is then applied for 20 s. **b** This causes the gel to become strongly adhered to the tissue. **c** When the gel-tissue pair is placed in the field with reversed polarity (E^+^T^−^G^+^E^−^), then within 10 s, the adhesion is lost and the gel can be detached from the tissue. The above photos are stills from Supplementary Movie 3.

We then set out to determine whether electroadhesion could be extended to soft materials other than gels. Specifically, could gels be electroadhered to animal tissues? For these studies, we worked with bovine tissues, obtained from a local butcher. Tissues were cleaned and prepared for our studies, as exemplified in Supplementary Fig. 2. Tissue samples were then tested along with the same QDM gels as above (Fig. 5). First, we tested a piece of bovine aorta, which is one of the largest arteries in an animal^24^. A rectangular strip (1.5 × 2.5 cm^2^) of the aorta was used along with a similar strip of the QDM gel. As shown in Fig. 5a, the gel and tissue are contacted with electrodes in the same orientation as before (E^+^G^+^T E^−^), with the cationic QDM gel (G^+^) connected to the positive electrode and the tissue (T) to the negative electrode. A DC voltage of 10 V is then applied for ~20 s, whereupon the gel becomes strongly adhered to the tissue (Fig. 5b). This suggests that the tissue behaves like an anionic gel, and hence we notate it as T^−^. No adhesion is observed if the reverse orientation (E^+^T^−^G^+^E^−^) of the field is used. Also, if the electroadhered gel-tissue pair is placed in the reverse orientation and the field is applied, the gel-tissue adhesion is reversed and the two can be easily separated, as in the gel-gel case (Fig. 5c). We conclude that the QDM gel can be reversibly electroadhered to the aorta. Note that the photos in Fig. 5 are from Supplementary Movie 3, which depicts the entire process.

Regarding the strength of electroadhesion between gel and tissue, a few points need to be clarified. First, when the QDM gel is contacted with the aorta in the absence of the field, we do find a weak adhesion, which we term ‘contact adhesion’. This adhesion is weak enough that the gel can be peeled off intact from the tissue by hand without much force (as shown in Supplementary Movie 3). In contrast, when the gel is electroadhered to the aorta, the gel cannot be peeled off intact by hand or lifted off from the aorta by using a scalpel (see the above movie). We thereby conclude that strong adhesion of the gel to the tissue is induced by the field and that this adhesion is much stronger than the contact adhesion between the two. We will quantify the strength of electroadhesion later in this paper.

Once electroadhesion of the QDM gel to the aorta was confirmed, we proceeded to test the same phenomenon with other classes of bovine tissue. In all cases, we cut a strip of tissue similar to that in Fig. 5 and tested it against a similar strip of QDM gel. First, we examined if there was “contact adhesion” when the strips of gel and tissue were pressed together without a field. The extent of adhesion (or lack thereof) was assessed using a subjective scale for adhesion strength (see Table 1). For this purpose, we attempted to detach the gel from the tissue and noted the ease with which this could be done. The results were then classified into: 0 = negligible, 1 = weak, 2 = moderate, 3 = strong, and 4 = very strong adhesion. For a given gel-tissue pair, the results for ‘contact adhesion’ provided the baseline. Next, we attempted to induce electroadhesion of the same gel-tissue pair using the same protocol as in Fig. 5 (i.e., using 10 V of DC, applied for 20 s). After the field was switched off, we again assessed the adhesion strength using the above 0–4 scale and compared the results to the baseline. The results for all types of tissue are presented in Table 1.

**Table 1:** Results of electroadhesion tests done with QDM gels and various bovine tissues.

Adhesion			No Adhesion		
Tissue (Bovine)	Adhesion Strength, Following Electroadhesion	Adhesion Strength Following Contact Adhesion	Tissue (Bovine)	Adhesion Strength, Following Electroadhesion	Adhesion Strength Following Contact Adhesion
Aorta (descending thoracic)	3	1–2	Heart	0–1	0
Cornea (inner layer)	3–4	0	Brain	0–1	0
Lung	2	0	Spleen	0–1	0
Cartilage (articular)	2	0	Fat	0	0
			Thymus	1–2	1
Tendon (transverse section)	3–4	0	Tendon (longitudinal section)	1–2	0
Skeletal muscle (neck, transverse section)	2–3	0	Skeletal muscle (neck, longitudinal section)	1	0
Skeletal muscle (cheek, transverse section)	2	0	Skeletal muscle (cheek, longitudinal section)	1–2	0

*N* = 3 for all samples. All electroadhesion tests done with 10 V DC, applied for 20 s.

Numerical scores represent strength of adhesion assessed on a scale of 0–4: 0—Negligible; 1—Weak; 2-Moderate; 3—Strong; 4—Very strong.

Electroadhesion was significant relative to contact adhesion only for the tissues listed in the left half of the table.

Table 1 (left half) indicates several tissues for which the strength of electroadhesion is much higher than the baseline case of contact adhesion. The largest contrast is in the case of the cornea from the eye, where the QDM gel shows negligible contact adhesion (0 on the scale), but very strong electroadhesion (~4 on the scale). Other tissues for which electroadhesion is clearly stronger and distinct relative to the baseline include the lung, the cartilage, and certain types of skeletal muscle. The case of the aorta, which was depicted above in Fig. 5 is one in which contact adhesion is not zero, but electroadhesion is clearly much stronger. Conversely, the right half of Table 1 lists the tissues for which electroadhesion is not significant under the conditions studied. In the case of the heart, the brain, the spleen, and fat tissue, there is no significant adhesion, either on contact or due to the field. In the case of the thymus, weak adhesion is observed due to the field, but this is not sufficiently distinguishable from contact adhesion. Tissues can be structurally complex, and the complexity is especially evident in our studies with tendon and skeletal muscle (bottom three entries in Table 1). If these tissues are cut in a longitudinal section, the samples do not exhibit significant electroadhesion; however if the same tissues are cut in a transverse section, electroadhesion is appreciable. Thus, there is significant anisotropy in the tissue structure, which also affects the results here. All in all, we conclude from Table 1 that cationic QDM gels can be electroadhered to some types of animal tissue.

Why does electroadhesion work with tissues? And why does it work only with some tissue types and not others? We made anionic counterparts to the QDM gel by copolymerizing AAm with an anionic monomer like sodium acrylate (SA). However, this gel could not be electroadhered to any tissues. Thus, in all the successful cases of electroadhesion we have found, the gel was cationic (i.e., QDM), which then implies that the tissue must be anionic. Animal tissues are expected to have a microstructure consisting of cells (either discrete or close-packed into clusters) embedded in a network of polymer chains, i.e., the extracellular matrix (ECM)^9,10^. The ECM tends to have different composition in different tissues. Two key proteins in the ECM are collagen and elastin. We attempted to find the percentage of each of these proteins in the tissues studied here^25–29^. These numbers are shown in Supplementary Table 1, which is divided into two halves similar to Table 1, with the tissues exhibiting electroadhesion on the left and those that do not on the right. In addition to the proteins, the water content in each tissue is also shown.

One observation from Supplementary Table 1 is that many (but not all) the tissues in the left half have a high collagen content. Collagen itself is a protein that has a net neutral charge at ambient pH^30,31^, and on its own would not impart anionic character to the tissue. However, collagen-rich tissues often are associated with protein-sugar hybrid polymers called glycosaminoglycans (GAGs), which are known to be strongly anionic^29,32^. The GAGs anchor cells to the ECM by attaching simultaneously to proteins on the cell surface as well as to the collagen fibers in the ECM.^32^ GAGs such as heparan sulfate have high affinity to collagen type I and III^32^ which are the main types of collagen in the aorta. Another observation from Supplementary Table 1 is that some types of collagen-rich ECMs also have high concentrations of elastin. Elastin is reported to be cationic at ambient pH, which allows it to bind to GAGs via electrostatic interactions. Collectively, in a tissue that contains collagen, GAGs, elastin, and other polymers, the overall composition of charged polymers will dictate the net charge of the tissue. It is possible that only tissues with a net anionic character will have a propensity to undergo electroadhesion (to cationic gels like QDM). Another factor could be the water content in the tissue; if this is too low (such as in the case of fat or brain tissue), the tissue may not exhibit electroadhesion.

An additional factor to consider is the ionic strength of the (fluid in the) tissue. Interactions between cationic and anionic polymers will be impacted by the ionic strength. In this regard, we have soaked the QDM gel and a representative tissue (aorta) in different fluids of biological relevance and then examined their adhesion. All these fluids are expected to have an ionic strength around 0.15 M. If soaked in whole blood (bovine), the gel and tissue electroadhere just as in their native state. When soaked in blood plasma (bovine) or in phosphate-buffered saline (PBS), the gel-tissue adhesion was initially weaker, but built up thereafter. By increasing the time over which the field was applied from 20 to 60 s, we were able to obtain significant adhesion between the gel and tissue in all cases.

We have also recorded the current *I* during electroadhesion experiments, which is reported for various pairs in Supplementary Fig. 3. Data in this figure are shown for the current density *j* (i.e., *I*/area of contact). We note that *j* seems to depend mainly on the ionic strength of the gel and tissue: for example, *j* is 52 mA/cm^2^ when two gels (G^+^ and G^−^) are electroadhered in their native state (i.e., after preparation with deionized water) but increases to 126 mA/cm^2^ when the same gels are soaked in PBS. Similar currents were observed even if gels of the same charge (e.g., two G^+^) were contacted, which is a case of no adhesion. In the case of gel-tissue experiments, *j* for various tissues are shown in Supplementary Fig. 3. For tissues that electroadhere, *j* varies from a low of 17 mA/cm^2^ for the lung to around 80 mA/cm^2^ for the aorta and cornea. For tissues that do not electroadhere, *j* is nearly zero for fat tissue, whereas it is 42 mA/cm^2^ for heart tissue. From these numbers, no clear relationship can be discerned between *j* and adhesion (or lack thereof). It should be mentioned that the *j* values we report correspond to the highest current recorded, which is near the start of the experiment. With time, the current drops to a steady-state that is 20–30% of the above values.

**Fig. 6:**
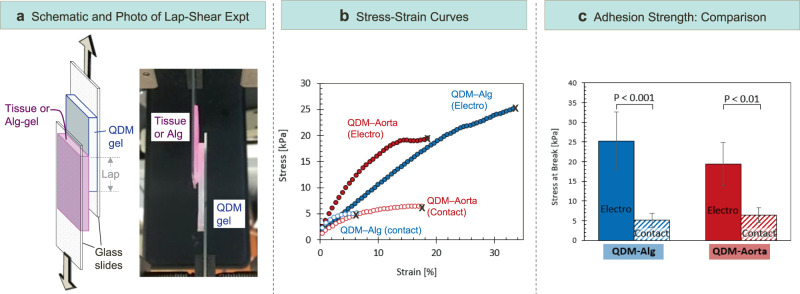
Adhesion strength measurements using the lap-shear protocol. **a** Schematic of the lap-shear experiment and a photo of an experiment in progress. Samples are first adhered over a lap region and then affixed to glass slides on their reverse sides using cyanoacrylate glue. Tension is then applied to the ends of the slides. **b** Stress vs. strain curves from lap-shear experiments for two sets of samples: gel-gel (QDM-Alg) and gel-tissue (QDM-aorta). Data are shown for the cases of electroadhesion and contact adhesion (control). The samples delaminate at the end of each curve, marked by an X. The stress at this point is a measure of the adhesion strength. **c** Adhesion strengths from the curves in (**b**) for the QDM-Alg and QDM-Aorta samples and for the two cases of electro- and contact adhesion. In each category, at least three samples were studied and the averages are plotted. Error bars correspond to standard deviations.

Next, we attempted to measure the gel-tissue adhesion strength and compare it to that for the gel-gel case. The measurements were done using the lap-shear test protocol, which is described in more detail in the Methods section.^33–35^ In this test, two rectangular samples are adhered to each other over a portion of their area, which is called the “lap” (Fig. 6a). The outer surfaces of the two samples are then stuck to glass slides using cyanoacrylate glue. The setup is then placed in the testing instrument, with each glass slide being gripped on its end by the jaws of the instrument. A tensile strain is then applied until failure occurs, and the magnitude of the stress-at-break is a measure of the adhesion strength. Stress vs. strain curves are presented in Fig. 6b for two sets of samples: a QDM gel adhered to an Alg gel, and the same QDM gel adhered to bovine aorta. For both cases, we ran the test first under “contact adhesion”, where the samples are pressed together without a field. Next, the two samples are electroadhered and the test is repeated. In both the gel-gel and the gel-tissue cases, the stress-strain curves for electroadhesion extend up to much higher stresses compared to contact adhesion (Fig. 6b), indicating the strong adhesion imparted by the electric field.

The adhesion strengths determined from the above curves are plotted in Fig. 6c. The strength of gel-gel (QDM-Alg) electro-adhesion is found to be ~25 kPa. For comparison, Asoh and Kikuchi measured the adhesion strength (using the same lap-shear technique) for a pair of cationic and anionic acrylamide-based gels and reported values around 10 kPa.^2^ For the electroadhered gel-tissue pair (QDM- aorta), the adhesion strength is about 20 kPa, which is comparable to that for the gel-gel case. In both cases, the strength of contact adhesion is much lower (~5 kPa). These measurements confirm our findings from earlier in the paper that, for both the gel-gel and gel-tissue cases, electroadhesion is substantially strong. One difference we noted was regarding the failure mode in the lap-shear experiment. In the gel-gel case, when failure occurred, it was generally a cohesive failure^33–35^, i.e., pieces of each gel were found to remain on the other. In the gel-tissue case, failure was also generally cohesive^33–35^, but whereas some gel remained stuck to the tissue, no tissue remained stuck to the gel. This difference could be because the tissue tested (aorta) was generally much stiffer than the QDM and Alg gels.

We also studied the adhesion strength as a function of time under the electric field, and the corresponding data are shown in Supplementary Fig. 4. These data are for QDM gels crosslinked with BIS (but not containing LAP nanoparticles) in contact with bovine aorta. The same lap-shear protocol as in Fig. 6 was used and the stress-at-break was used as a measure of adhesion strength. Gel-tissue pairs were placed for different times in an electric field generated by 10 V DC. The data reveal that sufficient electroadhesion (i.e., much higher than contact adhesion) develops within 10 s in the field. Subsequently, the adhesion strength tapers off to a constant value by about 20 s, and similar values are obtained with higher contact times (40 s). Thus, a time of 20 s in the field seems to be more than adequate to induce appreciable electroadhesion between gel and tissue. Similar data for adhesion strength as a function of contact time has been reported previously for gel-gel adhesion.^5^

### Electroadhesion for repairing cut or damaged tissue

**Fig. 7:**
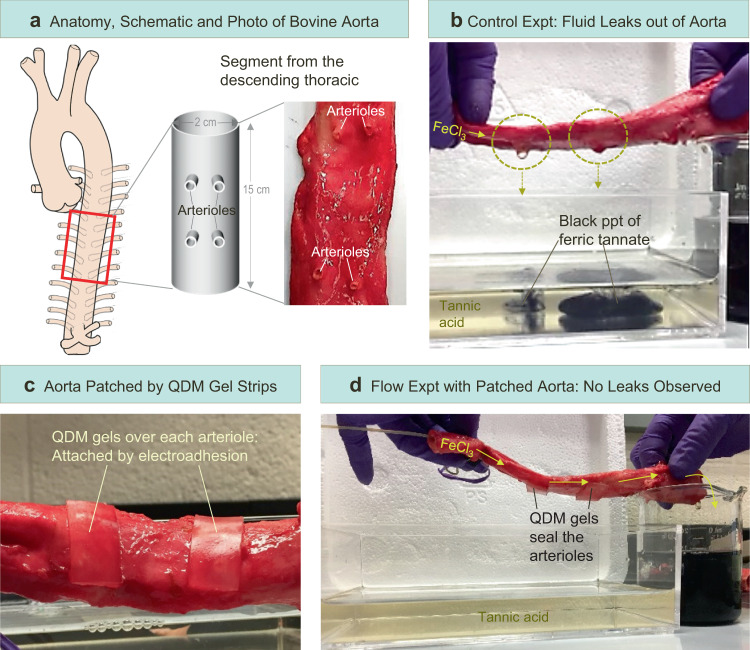
Electroadhesion of QDM gels to patch openings in the aorta. **a** The anatomy of the aorta, which is a large artery, is depicted on the left. A 15-cm long segment from the descending thoracic region of the aorta is used in the study. The segment is a hollow tube that has holes on its surface corresponding to arterioles (side branches), as shown both in the schematic and the photo. **b** When an aqueous solution of 0.1 wt% FeCl_3_ is pumped through the aorta, the fluid leaks out of the arterioles and falls into the bath containing tannic acid, whereupon a black precipitate of ferric tannate is formed. **c** Two QDM gel strips are electroadhered to the aorta so as to cover the arterioles. **d** When the FeCl_3_ solution is pumped through the patched aorta, no leaks are observed (the bath stays clear), and the fluid flows steadily into the beaker on the right. The entire process is depicted in Supplementary Movie 4.

Finally, we explored whether an electroadhered gel patch could seal cuts on a tissue, effectively mimicking a surgical repair. These studies are similar to those we had previously demonstrated with the anionic gel tube in Figs. 3 and 4, where cuts were sealed by a QDM gel patch. We again chose the cationic QDM gel, but this time the experiment involved a segment from the descending thoracic aorta of a cow, which was about 15 cm long and 2 to 2.5 cm in diameter (Fig. 7a). For the purposes of our experiment, we exploited the fact that the aorta has pairs of holes along its length (marked by arrows in Fig. 7a). These holes correspond to arterioles, which are small branches from the aorta that transport blood to various organs.^24^ If the aorta is used as a tube, fluid will leak out through the arterioles. This is shown by Fig. 7b, where we have replicated the test protocol from Fig. 3. A solution of 0.1% FeCl_3_ is pumped through the lumen of the aorta. The fluid leaks out through the arterioles and drips into the bath containing 0.1% tannic acid, whereupon a black precipitate of ferric tannate is instantly formed. (Note that the aorta was not submerged in the bath to avoid any reaction of the tissue with the tannic acid.)

Next, we made two rectangular patches of the QDM gel for the two pairs of arterioles in the aorta segment under study. We affixed the gel patches over the arterioles (one patch covers two adjacent arterioles) using electroadhesion (10 V, 20 s). The gels adhered tightly to the tissue, as can be seen in Fig. 7c. Thereafter, the flow of FeCl_3_ solution through the aorta was restarted. Figure 7d shows that there are no leaks through the arterioles, i.e., the holes remain sealed, allowing fluid to flow right through the aorta. The fluid is collected in a beaker containing 0.1% tannic acid at the end of the aorta. The black precipitate of ferric tannate is seen in the beaker but not in the water bath, thus confirming that there are no leaks through the tissue during the flow process. Supplementary Movie 4 depicts the entire process, including the initial leakage of fluid out of the arterioles; the sealing of leakage points by electroadhesion of QDM gel patches; and finally, the stable flow of fluid through the sealed aorta.

### Prospects for biomedical applications

Our results indicate the potential for electroadhesion to be useful in biomedical scenarios: it could enable surgical repairs in the future to be performed without the need for any sutures. Compared to current surgical adhesives^11–18^, an electroadhered gel patch provides a very robust and durable seal that persists indefinitely. Also, the unique feature of electroadhesion is that it develops on command when an external stimulus is applied. In case of a mistake, the adhesion can be reversed and the gel patch can be readily detached from the tissue. Subsequently, the patch can be reapplied on command. Many challenges will have to be addressed for this technique to be translated to the clinic. First and foremost, a better understanding of the adhesion mechanism is needed. Ideally, we should have the ability to electroadhere gels to any type of tissue, provided the gel is chosen carefully. The gels should also be biocompatible and not cause an adverse immune response in the body. Moreover, for many types of surgical repairs, it will be important for the gel to be biodegraded into benign products within a set number of days after the surgery. We believe biocompatibility and biodegradability are tractable problems because, in principle, QDM could be replaced with many other kinds of cationic gels.

Last, there is a question about applying electric fields and whether that would be safe in regard to a live animal. The voltage used here is 10 V DC, which on its own is not especially high^36–38^. Moreover, the field has to be turned on only for 20 s, which is a short enough time to avoid any adverse reaction. Incidentally, with regard to the voltage, we have found that electroadhesion of gels to tissues can be achieved even with voltages as low as 3 V but applied for a longer duration (~60–120 s). No adhesion was seen for voltages below 3 V for both gel-tissue and gel-gel systems, largely consistent with previous studies^2,5^. Conversely, with some of the tissues in Table 1 for which electroadhesion was unsuccessful with the current protocol (10 V for 20 s), a longer application time (e.g., for 60 s) could change the results. Thus, altering these parameters as well as the chemistry of the gel could well lead to strong electroadhesion with more of the tissue types listed in the table. These are aspects that will need to be studied carefully in the future.

### Summary and future outlook

In this study, we have demonstrated that the phenomenon of electroadhesion can be applied to new materials and geometries. We utilized cationic (QDM) gels and animal (bovine) tissues. Gel and tissue were brought into contact with each other and with electrodes in a E^+^G^+^ T^–^ E^–^ orientation (i.e., with the cationic gel G^+^ touching the positive electrode E^+^ and the tissue T^–^ the negative electrode E^–^). A DC voltage of 10 V was then applied for 20 s, whereupon the gel became strongly adhered to the tissue, with the adhesion persisting after the field was turned off. The strength of adhesion between QDM gel and bovine aorta, measured by lap-shear testing, was ~ 20 kPa. In addition to the aorta, electroadhesion also worked with the cornea, the lung, cartilage, and certain types of skeletal muscle and tendon. Only cationic gels could be electroadhered to tissues, which implied that the tissues had anionic character. Also, if the electroadhered gel-tissue pair was placed in a field with reversed polarity, the adhesion was lost and the two could be separated.

We then explored the possibility of using electroadhesion to seal cuts or tears in tubes. Initial experiments in this regard were done with tubes of anionic Alg gel as a model system. As an extreme case, two severed pieces of an Alg tube were joined using an electroadhered QDM gel strip that was flexible enough to encircle the tube while spanning the cut segments. In a similar manner, in the case of bovine aorta, QDM gels were electroadhered over openings in the tissue (corresponding to arterioles). In both cases, the electroadhered patches provided a robust and durable seal, allowing fluid to flow right through the lumen of the tubes. These studies raise the possibility of using electroadhesion to perform surgical repairs in the future. The use of strongly adhered gel patches could obviate the need for sutures or staples in many surgical procedures. The ability to achieve adhesion on command with an electric field, and moreover the ability to reverse the adhesion in case of an error, could enable surgeries to be done in a rapid, durable, and precise manner. Future work will address the challenges mentioned in the previous section before this technique could be used in clinical applications (such as gel biocompatibility and immune tolerance).

## Methods

### Materials

The following chemicals were from Sigma-Aldrich: the monomers acrylamide (AAm) and N,N′-methylenebis(acrylamide) (BIS), the initiator ammonium persulfate (APS), calcium chloride dihydrate (CaCl_2_) salt, tannic acid, sodium hydroxide, phosphate buffered saline (PBS) tablets, and the dye rhodamine B. The accelerant N,N,N′,N′-tetramethylethylene-diamine (TEMED) was from TCI America. The monomer N,N′-dimethylaminoethyl methacrylate, quaternary ammonium salt (QDM) was from MPD Chemicals. Two biopolymers were purchased from Sigma-Aldrich: alginate (Alg) (from brown algae, medium viscosity) and agarose (Type 1-A, low EEO, melting temperature ~88 °C). Laponite XLG nanoparticles (LAP) were a gift from Southern Clay Products. Cyanoacrylate-based glues (Gorilla Glue gel and Krazy Glue) and Rust-Oleum hydrophobic coating were purchased from The Home Depot. Deionized (DI) water was used in all experiments.

### Synthesis of alginate tubes

Alginate tubes were prepared by a variation of the method described by Gargava et al.^20^ First, a template of cylindrical agarose gel containing Ca^2+^ ions was prepared. For this, 2.5 wt% of agarose and 5 wt% of CaCl_2_ were added to DI water and heated above 80 °C until the agarose completely dissolved. The hot solution was then poured into a tube that was capped at one end. Upon cooling to room temperature, a solid (gel) cylinder of agarose was obtained. This cylinder was then placed in a solution of 2 wt% Alg for 12 min. During this time, Ca^2+^ ions diffuse out of the agarose, leading to an Alg gel around the cylindrical core. The final step was to dip this material in a 3 wt% CaCl_2_ solution for 20 min and then cut off the edges. The tube of Alg could then be slid off the agarose core. Alg tubes can be prepared over a range of dimensions using this method. For our purposes, we prepared the tubes in two typical dimensions by using agarose cores of different diameters and lengths: (a) 1 cm diameter and 10 cm length; and (b) 2 mm diameter and 60 cm length. Tubes were stored in a 1 wt% CaCl_2_ solution and dyed with 0.1 mM rhodamine B for contrast purposes. Typically, tubes were used within 24 h of preparation.

### Synthesis of QDM gels

Cationic QDM gels were prepared using the following protocol. First, DI water was degassed by bubbling nitrogen gas for 30 min. To assist with easy removal of the gels, Petri dishes used in gel preparation were coated with a spray of Rust-Oleum hydrophobic coating, then allowed to sit for 10 min, and thereafter wiped dry. Two variations of QDM gels were prepared: with and without LAP. For synthesis of QDM gel without LAP the following were combined: 1 M (1.4 g) AAm, 0.16 M (809 μL) of QDM solution, 0.019 M (0.06 g) BIS, 0.0088 M (0.04 g) APS and 0.01 M (30 μL) TEMED in 20 mL of degassed DI water. Next, the above monomer mixture was poured into a pre-coated Petri dish and maintained in a nitrogen environment for 3 h, whereupon the gel became fully polymerized. For synthesis of QDM gel with LAP, the first step was to add 1 wt% (0.2 g) of LAP particles to 20 mL degassed water and to stir until the particles were well-suspended (as ascertained by the sample appearing clear and homogeneous). Thereafter, the pH of the solution was lowered to 4.5 using 1 M HCl. Next, 0.16 M (809 μL) QDM was added dropwise to the LAP mixture followed by 1 M (1.4 g) AAm, 0.0095 M (0.03 g) BIS, 0.0088 M (0.04 g) APS and 0.01 M (30 μL) TEMED. When the pH was below 5, QDM was able to dissolve in the LAP suspension (without clumping). TEMED increased the pH back up to around 8.5. The above solution was placed in a pre-coated Petri dish and polymerized as before. After polymerization, gels were stored in a fridge and typically used within 24 h of preparation.

### Synthesis of SA gels

Anionic SA gels were prepared by a similar procedure as described above for QDM gels. In this case, the monomer solution contained 1.4 M (2 g) AAm, 0.11 M (0.2 g) SA, 0.019 M (0.06 g) BIS, 0.0088 M (0.04 g) APS and 0.01 M (30 µL) TEMED in 20 mL of degassed DI water. The above solution was poured into a pre-coated Petri dish and maintained in a nitrogen environment for 2 h. After polymerization, gels were stored in a fridge and typically used within 24 h of preparation.

### Tissue preparation protocol

All tissues were obtained ethically, immediately after slaughter from a local butcher. All experiments on tissues were conducted within 24 h of tissue harvest. When the tissues were first received, organs were typically encased in fat and other matrix material. For example, the aorta was surrounded with fat and connected to parts of the heart and lungs (see Supplementary Fig. 2). Thus, for experiments with the aorta, it had to be harvested and cleaned from the surrounding parts. The harvested aorta was then further segmented into smaller pieces for the electroadhesion experiments, as shown in the above figure. For many experiments, segments of tissue were sliced to a thickness of 0.3 ± 0.1 mm. The exceptions were in the cases of tissues that were naturally thin, such as the cornea.

### Adhesion experiments

A DC power source (Agilent, model E3612A) with a range of 0–60 V, 0–0.5 A was used for the electroadhesion experiments. The voltage was set to 10 V for most experiments. Graphite electrodes (from Saturn Industries) were cut to a size of 2 × 3 × 0.15 cm, and these were connected to the DC power source using alligator clips. The electrodes were placed on either side of the gel-gel pair or gel-tissue pair, as shown in Figs. 2 and 5. The gel strips were generally 2 mm in thickness, while the tissue strips were between 2 and 5 mm in thickness. Thus, the electric field strength across the gel-tissue sandwich was between 1.4 and 2.5 V/mm. For the gel-tissue experiments reported in Table 1, the following procedure was used. From a given batch obtained from the butcher, tissues of interest were harvested, and for a given tissue type, at least three tissue samples were prepared as described above. Three QDM gel strips were then prepared. First gel-tissue contact adhesion was measured, and then their electroadhesion. Two observers were used to independently rank the adhesion strength in each experiment on a scale of 0–4, where 0 = negligible, 1 = weak, 2 = moderate, 3 = strong, and 4 = very strong adhesion (this scale is also indicated at the bottom of Table 1). The average of both observers’ rankings was recorded for that experiment. Whenever in question, the second observer was blinded to the sample type so that their assessment was not biased. After three such trials with a tissue, the average of the readings was determined, and this is the one shown in Table 1.

### Pressure testing

The test setup is shown schematically in Fig. 5a. A peristaltic pump (Pharmacia-LKB-pump P-1) was used to pump a 0.1% FeCl_3_ solution through the Alg tube at a flow rate 5 mL/min. The tube was placed in a basin with a length of 15 cm, width of 5 cm and height of 5 cm. Openings were made on both sides to allow passage of the tube. The basin was filled with 0.1% tannic acid solution up to a height of 2 cm and the Alg tube (60 cm in length) was placed such that its middle portion was submerged in this solution (see Fig. 5a). Clamps were fixed at the bottom of the basin to control the path and location of the Alg tube in the basin. A pressure gauge (PRTemp 1000, from Madge Tech) was placed upstream of the Alg tube and the pressure was recorded in real-time (every 2 s) on a computer using the Madge Tech software. Pressure readings were obtained during flow in the tube before puncture, during puncture and following puncture repair by electroadhesion of a QDM gel patch. Burst pressures (for a patched tube) were determined by clamping shut the far end of the Alg tube and continuing flow into the tube, leading to pressure build up within the tube. The highest pressure recorded before the patch became dislodged was designated as the burst pressure. All measurements correspond to individual trials.

### Lap-shear testing

Lap-shear tests were conducted using an Instron Model 5565 instrument. Tests were done according to protocols recommended by the American Society for Testing and Materials (ASTM) which have been used in previous studies.^33–35^ Gels and tissues were cut into rectangular segments with dimensions of 1.5 × 4 cm. The QDM gel segment was 3 mm thick, the Alg segment was 1 mm thick and tissue segments were 2.5 ± 1 mm thick. Gel-gel and gel-tissue samples were electroadhered over a lap height of ~1.5 cm (see Fig. 6a). Following electroadhesion, the reverse sides of the gel and tissue were stuck securely to glass slides using cyanoacrylate glue. For securing the QDM and Alg gels to the glass slides, Krazy Glue was found to be the best and a 1 h cure time was used. For securing tissue to the same slides, Gorilla Glue was the best and a 2 h cure time was used (during this time, the tissue face exposed to air was covered by a piece of gauze soaked with PBS solution). The glass slides provided a hard and non-elastic backbone for the Instron to grip onto, which ensured that shear was applied on the lap area alone. The Instron was then used to elongate the sample at a rate of 10 mm/min, and the force was recorded during this process. At least three samples were tested for each of the categories in Fig. 6c, and the statistics were analyzed using the Student’s *t* test.

## Supplementary information

Supplementary Information

Supplementary Movie 1

Supplementary Movie 2

Supplementary Movie 3

Supplementary Movie 4

## Data Availability

The data that support the findings of this study are available from the corresponding author upon request.
