# Supplementary material for: Reversible electroadhesion of hydrogels to animal tissues for suture-less repair of cuts or tears
Source: Nat Commun. 2021 Jul 20;12:4419. doi: 10.1038/s41467-021-24022-x (PMC8292548; doi:10.1038/s41467-021-24022-x)
Supplement: Supplementary file 1 — Supplementary Information [file 41467_2021_24022_MOESM1_ESM.pdf]

Supplementary Information for:

Reversible Electroadhesion of Hydrogels to Animal Tissues for Suture-Less Repair of Cuts or Tears

Leah K. Borden, Ankit Gargava and Srinivasa R. Raghavan*
Department of Chemical & Biomolecular Engineering
University of Maryland, College Park, Maryland 20742, USA

*Corresponding author. Email: sraghava@umd.edu

Contents

Sup. Fig. 1: Pressure changes in tube before and after applying a gel patch by electroadhesion.

Sup. Fig. 2: Preparation of a sample of bovine aorta for electroadhesion experiments.

Sup. Fig. 3: Current density during electroadhesion experiments on gel-gel and gel-tissue pairs.

Sup. Fig. 4: Gel-tissue adhesion strength as a function of time in the electric field

Sup. Table 1: Collagen, elastin, and water content in various tissues.

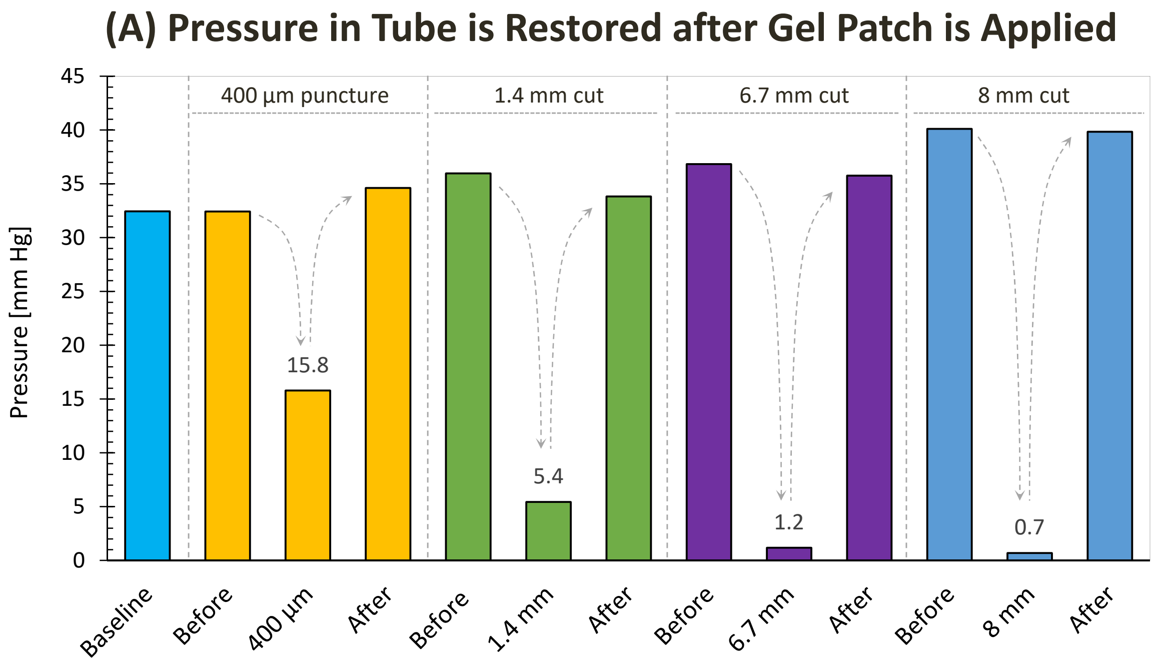

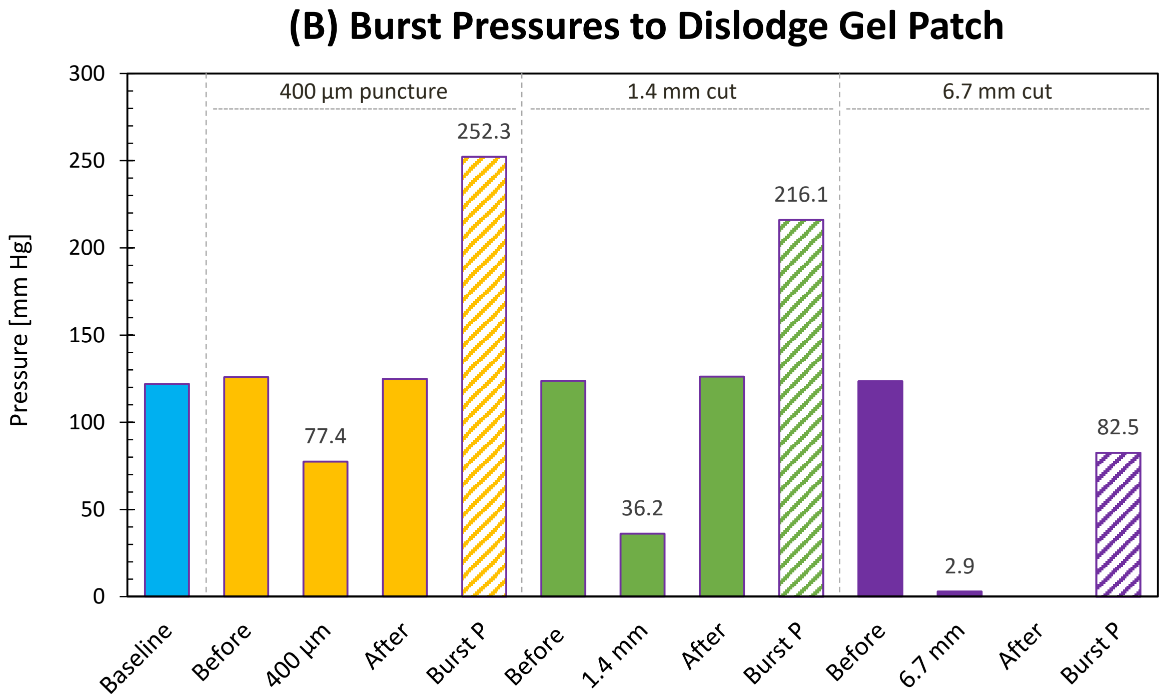

Supplementary Figure 1. Pressure changes in tube before and after applying a gel patch by electroadhesion. (A) Pressure readings before a puncture/cut is made in the wall of an alginate (Alg) tube and after the cut is sealed by electroadhesion of a QDM gel patch. Data are shown for different cut sizes, and for each case, the three bars are the readings for flow (i) before cut (baseline); (ii) when cut is made and not sealed; and (iii) after cut is sealed. In all cases, the pressure drops as fluid leaks out through the cut, but returns to baseline values once the cut is sealed. (B) For different cut sizes, data are further shown for the burst pressure required to dislodge the QDM gel patch from the tube wall. For these experiments, the baseline pressures are higher than in (A). Note that the burst pressure far exceeds the baseline pressure for small cut sizes.

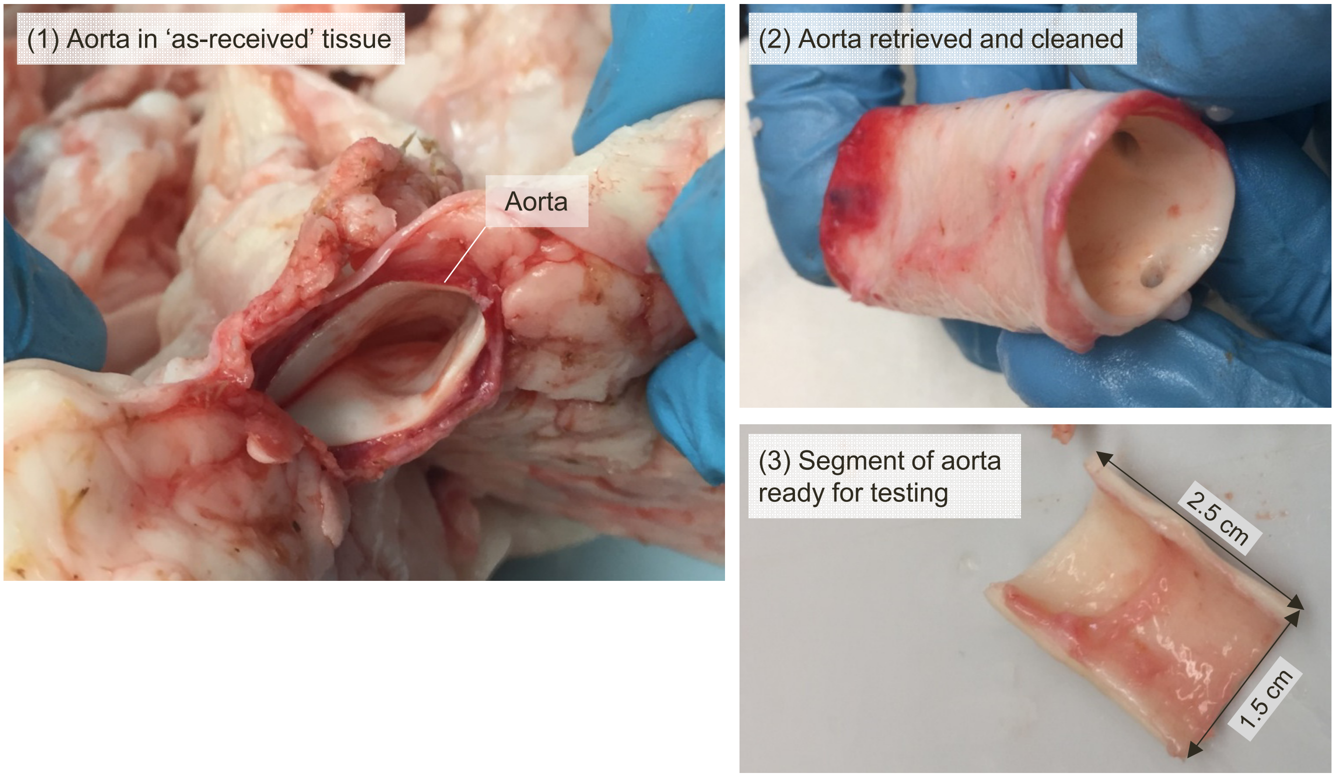

Supplementary Figure 2. Preparation of a sample of bovine aorta for electroadhesion experiments. (1) As recieved, in the "raw" state, the aorta is encased in fat, and connected to other tissues. (2) The aorta is removed from the surrounding tissues and the fat encasings. (3) A segment of the aorta is cut to the desired size, comparable to the size of a gel segment. This is used for the electroadhesion experiments.

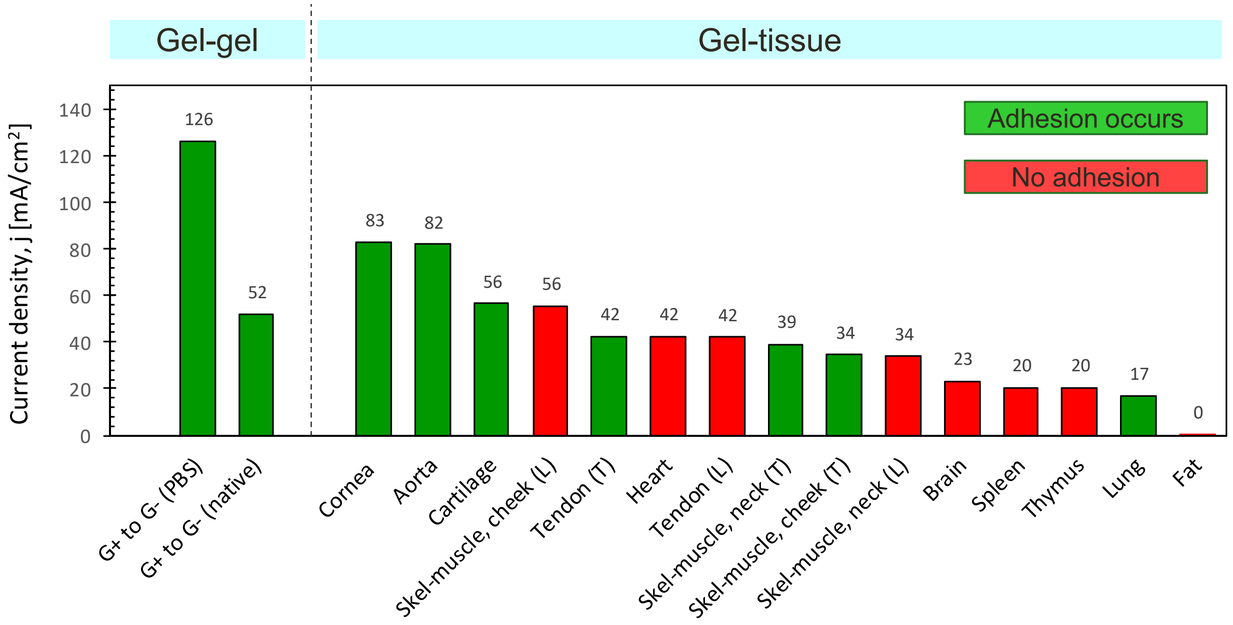

Supplementary Figure 3. Current density during electroadhesion experiments on gel-gel and gel-tissue pairs. All pairs were placed in an electric field of 10 V DC that was applied for 20 s. In the gel-gel cases, a cationic QDM gel (G^+) was contacted with an anionic SA gel (G^-). In the gel-tissue cases, the cationic QDM gel (G^+) was contacted with various tissues. In all experiments, the current I starts high and decreases with time. The highest recorded current is used to calculate the current densities j shown in the plot (note: $j = I$ / area of contact). The area of contact ranged between 1.6 and 2.4 cm^2 for the various tissues. No obvious correlation is seen between j and the occurrence of electroadhesion (refer to Table 1 in the text). The data for gel-gel pairs in their native state (i.e., as prepared in deionized water) or after soaking in PBS demonstrate that j mainly depends on the ionic strength of the fluid.

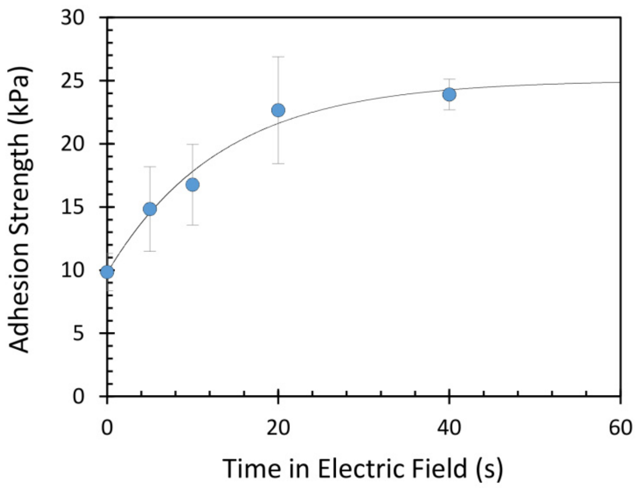

Supplementary Figure 4. Gel-tissue adhesion strength as a function of time in the electric field. Adhesion between QDM gels and aorta strips were measured after different durations of exposure to the field (10 V DC). The lap-shear technique was used and the stress-at-break was used to quantify the adhesion strength. The plot shows that electroadhesion develops within ~ 10 s in the field and the adhesion strength saturates by about 20 s. The data shown are averages (across at least three samples) and the error bars correspond to standard deviations. The line through the data is a guide to the eye.

Supplementary Table 1. Collagen, elastin, and water content of tissues that do and do not exhibit electroadhesion.

Adhesion				No Adhesion			
Tissue (Bovine)	Percent Collagen	Percent Elastin	Percent Water	Tissue (Bovine)	Percent Collagen	Percent Elastin	Percent Water
**Aorta**	**23**	**40**	**84-87**	**Heart**	**3**	**0**	**74**
**Cornea**	**70**		**78**	**Brain**	**0.2**	**0**	**73**
**Lung**	**11**	**5**	**84**	**Spleen**	**3**	**5**	**79**
**Cartilage**	**58**		**80**	**Fat**		**9**	**50**
				**Thymus**			
**Tendon** (transverse section)	**85**	**5**	**55-70**	**Tendon** (longitudinal section)	**85**	**5**	**55-70**
**Skeletal muscle** (transverse section)	**2-7**	**0**	**80**	**Skeletal muscle** (longitudinal section)	**2-7**	**0**	**80**

Values correspond to bovine tissue unless otherwise noted below. Values rounded to nearest integer.

Notes:

a. Aorta: Collagen and elastin compositions of aorta,[1] water content.[2]

b. Cornea: Collagen composition,[3] elastin percent values not given,[4] Water content.[5]

c. Lung: Collagen and elastin compositions of rat lung,[1] water content.[6]

d. Cartilage: Collagen composition of human cartilage,[7] elastin percent values not given,[8] water content.[9]

e. Tendon: Collagen and elastin compositions of tendon,[1] water content.[10]

f. Skelatel muscle: Collagen and elastin compositions of skeletal muscle,[11] water content.[6]

g. Heart: Collagen and elastin compositions of heart,[1] water content.[6]

h. Brain: Collagen and elastin compositions of rat brain,[1] water content.[6]

i. Spleen: Collagen and elastin compositions of spleen,[1] water content.[6]

j. Fat: Collagen percent values not given,[12] elastin composition of human adipose (fat) tissue,[13] water content.[6]

k. Thymus: Collegen percent values not given,[14] no data on elastin and water content available in literature.

Supplementary References

[1] Neuman, R. E. & Logan, M. A. The determination of collagen and elastin in tissues. *J. Biol. Chem.* **186**, 549-556 (1950).

[2] Wells, S. M. & Walter, E. J. Changes in the Mechanical Properties and Residual Strain of Elastic Tissue in the Developing Fetal Aorta. *Ann Biomed Eng* **38**, 345-356 (2010).

[3] Tseng, S. C. G., Smuckler, D. & Stern, R. Comparison of collagen types in adult and fetal bovine corneas. *J. Biol. Chem.* **257**, 2627-2633 (1982).

[4] Feneck, E. M., Lewis, P. N., Ralphs, J. & Meek, K. M. A comparative study of the elastic fibre system within the mouse and human cornea. *Exp Eye Res* **177**, 35-44 (2018).

[5] Taylor, Z. D. et al. THz and mm-Wave Sensing of Corneal Tissue Water Content: Electromagnetic Modeling and Analysis. *Ieee T Thz Sci Techn* **5**, 170-183 (2015).

[6] Mitchell, H. H., Hamilton, T. S., Steggerda, F. R. & Bean, H. W. The Chemical Composition of the Adult Human Body and Its Bearing on the Biochemistry of Growth. *J Biol Chem* **158**, 625-637 (1945).

[7] Yin, J. H., Xia, Y. & Lu, M. Concentration profiles of collagen and proteoglycan in articular cartilage by fourier transform infrared imaging and principal component regression. *Spectrochim. Acta A* **88**, 90-96 (2012).

[8] Mansfield, J. et al. The elastin network: its relationship with collagen and cells in articular cartilage as visualized by multiphoton microscopy. *J Anat* **215**, 682-691 (2009).

[9] Sophia Fox, A. J., Bedi, A. & Rodeo, S. A. The basic science of articular cartilage: structure, composition, and function. *Sports health* **1**, 461-8 (2009).

[10] Aparecida de Aro, A., Vidal Bde, C. & Pimentel, E. R. Biochemical and anisotropical properties of tendons. *Micron* **43**, 205-14 (2012).

[11] Bendall, J. R. Elastin content of various muscles of beef animals. *J. Sci. Food Agric.* **18**, 553-558 (1967).

[12] Divoux, A. et al. Fibrosis in human adipose tissue: composition, distribution, and link with lipid metabolism and fat mass loss. *Diabetes* **59**, 2817-25 (2010).

[13] Spencer, M. et al. Adipose tissue extracellular matrix and vascular abnormalities in obesity and insulin resistance. *The Journal of clinical endocrinology and metabolism* **96**, E1990-8 (2011).

[14] Pearse, G. Normal structure, function and histology of the thymus. *Toxicologic pathology* **34**, 504-14 (2006).
